# Central nervous system metastases in breast cancer patients with germline *BRCA* pathogenic variants compared to non-carriers: a matched-pair analysis

**DOI:** 10.1186/s12885-024-11975-7

**Published:** 2024-02-16

**Authors:** Matan Ben-Zion Berliner, Shlomit Yust-Katz, Inbar Lavie, Yael Goldberg, Inbal Kedar, Rinat Yerushalmi

**Affiliations:** 1 Breast cancer Unit, Davidoff Cancer Center, Rabin Medical Center–Beilinson Hospital, Petach Tikva, Israel; 2 Neuro-Oncology Unit, Davidoff Cancer Center, Rabin Medical Center–Beilinson Hospital, Petach Tikva, Israel; 3 The Raphael Recanati Genetics Institute, Rabin Medical Center–Beilinson Hospital, Petach Tikva, Israel; 4 Faculty of Medicine, Tel Aviv University, Tel Aviv, Israel

**Keywords:** Breast cancer, Central nervous system (CNS) metastasis, *BRCA* mutation, Leptomeningeal metastasis

## Abstract

**Background:**

Breast cancer is a common cause for central nervous system (CNS) metastasis, resulting in a significant reduction in overall survival. Germline pathogenic variants (PVs) in *BRCA1/2* are the most common genetic risk factor for breast cancer, associated with poor prognostic factors. This study sought to explore the patterns and outcome of CNS metastases in breast cancer patients with germline PVs in *BRCA*1/2 genes.

**Methods:**

A retrospective cohort of 75 breast cancer patients with known *BRCA*1/2 mutation status, who were diagnosed with CNS metastases in 2006–2021. Histopathology, characteristics of CNS disease, treatments, and survival were compared between *BRCA*1/2 carriers (*n* = 25) and non-carriers (*n* = 50), using propensity score matching (1:2 ratio) to control for the possible influence of tumor receptor status (ER, PR, HER2) and patient age. Pearson chi-square or Fisher exact test and Kaplan-Meier survival curves with log-rank test were used for statistical analyses.

**Results:**

Patients with PVs in *BRCA1/2* had more high-grade tumors (88% vs. 68%, *P* = 0.060), were younger at CNS disease diagnosis (median 46.69 vs. 55.02 years, *P* = 0.003) and had better ECOG performance status (ECOG PS 0 in 20% vs. 2%, *P* = 0.033), but without significant differences in systemic or CNS-directed treatment approaches. *BRCA*1/2 mutation was associated with a higher rate of temporal lobe involvement (52% vs. 26%, *P* = 0.026) and leptomeningeal spread (40% vs. 20%, *P* = 0.020). Survival after diagnosis of CNS disease was shorter (median 8.03 vs. 28.36 months, *P* < 0.0001), with no significant differences in time to development of CNS metastases or overall-survival.

**Conclusion:**

Patients with CNS metastatic breast cancer and PVs in *BRCA1/2* showed a higher rate of leptomeningeal and temporal lobe involvement, and a shorter survival with CNS disease. To the best of our knowledge, this is the first study suggesting an exclusive impact of germline *BRCA*1/2 mutations in CNS metastatic breast cancer.

## Background

Breast cancer is a common cause for central nervous system (CNS) metastasis, diagnosed in 10–16% of patients [1], with higher rates of up to 30–50% among patients with human epidermal growth factor 2 (HER2) overexpression [2] or triple-negative disease [3]. Other risk factors for CNS metastatic disease include young age at breast cancer diagnosis, tumors with high histologic grade, and the presence and number of distant metastatic sites [4–6]. CNS disease is diagnosed at a median time of 16–54 months after diagnosis of breast cancer, usually due to clinical symptoms, as there is currently no evidence proving the benefit of routine screening or prophylactic treatment [7–9]. The occurrence of CNS metastases results in a significant reduction in overall survival, with a median of 16–25 months from diagnosis of CNS disease to death [7, 8, 10].

A genetic risk factor can be identified in 5–15% of breast cancers, most commonly mutations in *BRCA*1 or *BRCA*2, tumor suppressor genes associated with DNA damage repair. Germline pathogenic variants (PVs) in *BRCA1/2* are associated with a higher risk for certain malignancies, such as breast, ovarian, prostate, and pancreatic cancers. In a meta-analysis of 10 studies, there was a 57% risk for developing breast cancer before the age of 70 in women with *BRCA*1 pathogenic variants, and 49% in women with *BRCA*2 pathogenic variants [11–14]. Data also suggest a relationship between *BRCA1/2* mutations and poor prognostic factors, such as younger age at breast cancer diagnosis, high-grade tumors, and for *BRCA*1 mutation - a higher rate of triple-negative disease [14–16].

Little is known about the association between the loss of function of *BRCA1/2* genes and the patterns of CNS involvement in breast cancer. Studies on *BRCA*1-mutated breast cancer showed a higher and earlier incidence of brain metastasis, also after controlling for other known prognostic factors such as age, disease stage, and tumor receptor status (ER, PR, HER2) [17]. Limited data also suggested a higher rate of brain metastases in breast cancer with *BRCA*2 mutation, independent of the tumor histologic subtype [16]. This study sought to assess the patterns and outcome of CNS metastases in breast cancer patients with PVs in *BRCA*1/2 in comparison to *BRCA* wild-type (*BRCA*-WT) patients.

## Methods

### Study population

The database of *Davidoff Center for the Treatment and Research of Cancer* at *Rabin Medical Center* was searched for consecutive patients with breast cancer who were diagnosed with CNS metastases between July 2006 and August 2021. Inclusion criteria for the study were age ≥ 18 years, female sex, known *BRCA* mutation status according to standard genetic testing, and availability of contrast-enhanced computed tomography (CT) or magnetic resonance imaging (MRI) scans of the brain showing the pattern of CNS involvement. Patients were excluded if they had a second malignancy other than breast cancer that was discovered before the diagnosis of brain metastasis.

### Study variables

The following data were collected from the medical records of the eligible patients: age at diagnosis of breast cancer, age at diagnosis of CNS involvement, histopathological factors of the tumor (histology, grade, hormone receptor and HER2 status), treatments administered before and after CNS involvement, characteristics of CNS involvement, overall survival, time to diagnosis of CNS involvement, and survival after diagnosis of CNS disease. CT/MRI imaging scans were analyzed for type, location, number, dissemination, and local complications of the brain deposits. The diagnosis of leptomeningeal disease was based on radiographic findings and/or cerebrospinal fluid cytology showing malignant cells. *BRCA1/2* mutation status was assessed after genetic counseling at *the Raphael Recanati Genetic Institute of Rabin Medical Center*, based on the patient’s ethnic origin, family history, tumor subtype, and patient desire. Genetic tests included founder *BRCA* mutations testing or gene sequencing either by Sanger or Next generation sequencing. Only patients with pathogenic or likely pathogenic variants were classified as carriers. Patients with variants of uncertain significance (VUS) were not considered positive for mutation.

We performed a 1:2 matched-pair analysis of 25 patients with documented *BRCA* mutations (*BRCA*1: *n* = 19, *BRCA*2: *n* = 6), compared with 50 *BRCA*-WT matched controls. A systemic manual match was performed in relation to patient age at breast cancer diagnosis and tumor receptor status (ER, PR, HER2). From the 50 patients in the control group (*BRCA*-WT), the two patients with matches in the highest percentage of the aforementioned variables were chosen as best matches for each *BRCA* mutation patient. Each case was first matched for age, followed by ER PR and HER2 expression. Age matches were within 5 years in most cases, and ER and HER2 expression were matched in all cases. Estrogen-receptor (ER) status and progesterone-receptor (PR) were considered positive if > 1% of cells were found to have the receptor on immunohistochemistry (IHC) study. HER2 status was assessed by positive cell staining for the protein; scores were defined as follows: 0 or + 1 as negative, + 2 as equivocal, and + 3 as positive. In cases of IHC + 2, HER2 gene amplification was performed by fluorescence in situ hybridization (FISH) to confirm the status.

### Statistical analysis

Patients were compared for clinical characteristics using Pearson chi-square test, and Fisher exact test was used when > 20% of the cells had an expected count of < 5. Kaplan-Meier survival curves with log-rank test were used to compare three time periods: overall survival from breast cancer diagnosis, time from breast cancer diagnosis to development of CNS metastasis, and survival after the development of CNS metastasis. Statistical analyses were performed with SPSS version 26. Plots were generated using the survminer R computational package. Significance level (*P*-value) < 0.05 with a corresponding confidence level (confidence interval, CI) of 95% were chosen to define for statistical significance.

The study was approved by the local IRB of Rabin Medical Center.

## Results

### Study population comparison prior to CNS metastasis diagnosis

Clinical, pathological, and treatment characteristics prior to CNS metastasis diagnosis are shown in Table 1. Mean age at breast cancer diagnosis was 49.0 ± 9.4 years in patients without *BRCA* PVs (range 25.7–66.6), as compared to 39.4 ± 10.3 years in patients with *BRCA* PVs (range 37.1–58.7), *P* < 0.0001. Eastern Cooperative Oncology Group (ECOG) performance status (PS) at diagnosis of breast cancer was ≤2 in all patients, regardless of *BRCA* mutation status. The most common histological subtype in both groups was infiltrating ductal carcinoma (IDC). Tumor grade was intermediate-high (grade 2–3) in all patients, with more high-grade tumors among *BRCA1/2* carriers (88% vs. 68%, *P* = 0.060).

**Table 1:** Clinical and pathological factors and treatment characteristics prior to CNS metastasis diagnosis according to *BRCA* mutation status- carriers (*BRCA* 1/2 Mt) vs non-carriers (*BRCA* non-Mt)

Characteristics	*BRCA* non-Mt (*n* = 50)	*BRCA* 1/2 Mt (*n* = 25)		Total (*n* = 75)	*P-value*
**Tumor receptor status**		***BRCA*1 (*n* = 19)**	***BRCA*2 (*n* = 6)**		–
ER pos HER2 pos	10 (20%)	2 (10.5%)	3 (50%)	15 (11.25%)	
ER pos HER2 neg	20 (40%)	7 (36%)	–	27 (20.25%)	
ER neg HER2 pos	4 (8%)	2 (10.5%)	–	6 (4.5%)	
Triple-negative	16 (32%)	8 (42%)	3 (50%)	27 (20.25%)	
**Age at breast cancer diagnosis (yr)**					
Median	50	38.35		45.47	< 0.0001
Mean ± SD	49.04 ± 9.37	39.44 ± 10.25		45.84	
Range	25.68–66.64	37.13–58.68		25.67–66.63	
**ECOG PS at breast cancer diagnosis**^a^					
ECOS PS 0	35 (70%)	21 (84%)		56 (74.7%)	0.406
ECOG PS 1	10 (20%)	3 (12%)		13 (17.3%)	
ECOG PS 2	5 (10%)	1 (4%)		6 (8%)	
ECOG PS 3	0	0		0	
ECOG PS 4	0	0		0	
**Histology**					
IDC	44 (88%)	24 (96%)		68 (90.7%)	0.262
ILC	6 (12%)	1 (4%)		7 (9.3%)	
**Tumor grade**					
Low (Grade 1)	0	0		0	0.060
Intermediate (Grade 2)	16 (32%)	3 (12%)		19 (25.3%)	
High (Grade 3)	34 (68%)	22 (88%)		56 (74.7%)	
**Initial local treatment for breast cancer**^b^					
Lumpectomy+radiation	22 (44%)	13 (52%)		35 (46.7%)	0.611
Mastectomy	15 (30%)	8 (32%)		23 (30.7%)	
No treatment	13 (26%)	4 (16%)		17 (22.7%)	
**Axillary surgery**					
SLNBx	30 (60%)	16 (64%)		46 (61.3%)	0.330
ALND	7 (14%)	5 (20%)		12 (16%)	
None	13 (26%)	4 (16%)		17 (22.7%)	
**Risk-reducing mastectomy**					
Yes	2 (4%)	3 (12%)		5 (6.7%)	0.190
No	48 (96%)	22 (88%)		70 (93.3%)	
**Gonadal ablation**					
Prophylactic BSO	6 (12%)	9 (36%)		15 (20%)	0.027
Therapeutic BSO	5 (10%)	5 (20%)		10 (13.3%)	
Ablative gonadal RT	1 (2%)	0		1 (1.3%)	
None	38 (76%)	11 (44%)		49 (65.3%)	
**Pathological tumor stage (size) (pT)**^c^					
**T1**	24 (48%)	13 (52%)		37 (49.3%)	0.515
T2	12 (24%)	6 (24%)		18 (24%)	
T3–4	1 (2%)	2 (8%)		3 (4%)	
NA/unknown	13 (26%)	4 (16%)		17 (22.7%)	
**Pathological nodal status (pN)**^c^					
N0	19 (38%)	10 (40%)		29 (38.7%)	0.519
N1	9 (18%)	7 (28%)		16 (21.3%)	
N2	6 (12%)	4 (16%)		10 (13.3%)	
N3	3 (6%)	0		3 (4%)	
NA/unknown	13 (26%)	4 (16%)		17 (22.7%)	
**Disease spread at diagnosis**					
Local disease	37 (74%)	21 (84%)		58 (77.3%)	0.330
Metastatic disease	13 (26%)	4 (16%)		17 (22.7%)	
**Chemotherapy-local disease (Neo-adj/ Adj)**					
Anthracycline based	1 (2%)	0		1 (1.3%)	0.285
Anthracycline+	18 (36%)	15 (60%)		33 (44%)	
Taxane based					
Taxane-based	9 (18%)	2 (8%)		11 (14.7%)	
Non Taxane-non	10 (20%)	5 (20%)		15 (20%)	
Anthracycline^d^					
None	12 (24%)	3 (12%)		15 (20%(	
**Hormonal therapy- local disease (Neo-adj/ Adj)**					
Tamoxifen	11 (22%)	6 (24%)		17 (22.7%)	0.523
AI	3 (6%)	4 (16%)		7 (9.3%)	
Tamoxifen followed by AI	6 (12%)	3 (12%)		9 (12%)	
None	30 (60%)	12 (48%)		42 (56%)	
**anti-HER2- local disease (Neo-adj/ Adj)**					
Trastuzumab	4 (8%)	2 (8%)		6 (8%)	0.598
Trastuzumab+Pertuzumab	2 (4%)	0		2 (2.7%)	
TDM1	0	0		0	
None	44 (88%)	23 (92%)		67 (89.3%)	
**Chemotherapy-M1 non- CNS**					
Anti-microtubule^e^	15 (30%)	6 (24%)		21(28%)	0.489
Platinum based	1 (2%)	2 (8%)		3 (4%)	
Anthracycline based	4 (8%)	3 (12%)		7 (9.3%)	
Capecitabine	11 (22%)	7 (28%)		18 (24%)	
None	19 (38)	7 (28%)		26 (34.7%)	
**Hormonal therapy- M1 non-CNS**					
Tamoxifen	0	1 (4%)		1 (1.3%)	0.506
AI^f^	2 (4%)	3 (12%)		5 (6.7%)	
Tamoxifen followed by AI	2 (4%)	0		2 (2.7%)	
Fulvestrant	5 (10%)	3 (12%)		8 (10.7%)	
Anti-CDK4/6^g^+/− AI/ Fulvestrant	10 (20%)	4 (16%)		14 (18.7%)	
None	31 (62%)	14 (56%)		45 (60%)	
**anti-HER2- M1 non-CNS**					
Trastuzumab	4 (8%)	0		4 (5.3%)	0.282
Trastuzumab+Pertuzumab	5 (10%)	6 (24%)		11 (14.7%)	
TDM1	1 (2%)	1 (4%)		2 (2.7%)	
TKI based^h^	6 (12%)	4 (16%)		10 (13.3%)	
None	34 (68%)	14 (56%)		48 (64%)	
**Immunotherapy pre-CNS metastasis**^i^					
Yes	2 (4%)	0		2 (2.6%)	0.598
No	48 (96%)	25 (100%)		73 (97.3%)	
**No. treatment lines pre- CNS metastasis**					
No treatment for non- CNS metastasis	12 (24%)	2 (8%)		14 (18.7%)	0.269
1st line	19 (38%)	7 (28%)		26 (34.7%)	
2nd line	6 (12%)	5 (20%)		11 (14.7%)	
3rd line	7 (14%)	6 (24%)		13 (17.3%)	
4th line or later	6 (12%)	5 (20%)		11 (14.7%)	
**First site of systemic metastatic spread**					
Visceral	17 (34%)	10 (40%)		27 (36%)	0.843
Bone	6 (12%)	2 (8%)		8 (10.7%)	
CNS	8 (16%)	2 (8%)		10 (13.3%)	
Simultaneous multiple non-CNS sites	15 (30%)	9 (36%)		24 (32%)	
Simultaneous CNS + non-CNS	4 (8%)	2 (8%)		6 (8%)	
**No. of distant non-CNS metastatic sites**					
1 site	6 (12%)	2 (8%)		8 (10.7%)	0.969
2 sites	15 (30%)	8 (32%)		23 (30.7%)	
3–4 sites	22 (17.1%)	11 (44%)		33 (44%)	
5–6 sites	3 (6%)	2 (8%)		5 (6.7%)	
None	4 (8%)	2 (8%)		6 (8%)	

Values are n(%) unless otherwise indicated

*ECOG* Eastern Cooperative Oncology Group, *PS* Performance status, *IDC* Infiltrating ductal carcinoma, *ILD* Infiltrating lobular carcinoma, *SLNBx* Sentinel lymph node biopsy, *ALND* Axillary lymph node dissection, *BSO* Bilateral salpino-oophorectomy, *RT* Irradiation, *CNS* Central nervous system, *Neo-adj* Neo-adjuvant treatment, *Adj* Adjuvant treatment, *AI* Aromatase inhibitor, *HER2* Human epidermal growth factor receptor, *TDM1* Ado-trastuzumab emtansine, *M1* Metastatic disease, *CDK* Cyclin-dependent kinase, *TKI* Tyrosine-kinase inhibitor

^a^ECOG Performance Status, as published in: *Oken, M.M., Creech, R.H., Tormey, D.C., Horton, J., Davis, T.E., McFadden, E.T., Carbone, P.P. Toxicity and response criteria of the Eastern Cooperative Oncology Group. Am J Clin Oncol 5:649–655, 1982*

^b^To test the variable, None/Not done was removed, thus, only the applicable categories were compared

^c^According to American Joint Committee on Cancer (AJCC), 7th edition, 2010

^d^Non Taxane-non Anthracycline- including: CMF (cyclophosphamide, methotrexate, 5-Fluorouracil), CEF (cyclophosphamide, epirubicin, 5-Fluorouracil), Adjuvant Capecitabine (non-*BRCA* Mt: *n* = 2, *BRCA* Mt: *n* = 2)

^e^Anti-microtubule based therapy- including: Taxane (Paclitaxel, Docetaxel), Vinorelbine, Eribulin

^f^Aromatase inhibitor- including: non-steroidal (Letrozole, Anastrozole)

^g^Anti-CDK4/6 based therapy- including: Palbociclib (non-*BRCA* Mt: *n* = 9, *BRCA* Mt: *n* = 4), Ribociclib (non-*BRCA* Mt: *n* = 1), Abemaciclib (*n* = 0)

^h^TKI based therapy- including: Lapatinib (non-*BRCA* Mt: *n* = 5, *BRCA* Mt: *n* = 4), Neratinib (non-*BRCA* Mt: *n* = 1), Tucatinib (*n* = 0)

^i^Immunotherapy pre-CNS metastasis- including: Pembrolizumab (non-*BRCA* Mt: *n* = 1), Atezolizumab (non-*BRCA* Mt: *n* = 1)

Local treatment modalities were similar in both groups, with most patients undergoing breast lumpectomy and local irradiation therapy (46.7% of all patients) or mastectomy (30.7% of all patients), with sentinel lymph node biopsy (SLNBx) or axillary lymph node dissection (ALND) according to disease stage and clinical characteristics, and there were no significant differences in local treatment modalities in relation to *BRCA1/2* status. Double mastectomy with prophylactic removal of the contralateral breast was preformed according to individual patient decision and risk factors - in 12% of patients with *BRCA*1/2 PVs and in 4% with *BRCA*-WT, with no statistically significant difference (*P* = 0.190).

In the *BRCA* mutation group, 36% of patients underwent bilateral salpingo-oophorectomy (BSO), as compared to 12% in the *BRCA*-WT group (*P* = 0.027). Several patients had BSO or ablative gonadal irradiation for therapeutic purposes, to allow for hormonal suppression.

There were no significant differences in the tumor pathological T stage or N stage in regard of *BRCA* status. Metastatic disease was documented at the initial presentation in 22.7% of all patients, regardless of *BRCA* status.

In both groups, most of the patients (44% of all patients) received anthracycline and taxane-based treatments before the diagnosis of metastatic disease. Two patients from each group with triple-negative disease received capecitabine in the adjuvant setting for non-pathological complete response in surgery. Hormone-sensitive tumors were treated with endocrine-directed treatments, tamoxifen and/or aromatase inhibitors, and pre-menopausal patients also received luteinizing hormone-releasing hormone (LHRH) analogs. HER2-positive tumors were treated with HER2-directed treatments, including trastuzumab alone or in combination with pertuzumab as part of the treatment for local disease, in combination with chemotherapy and as maintenance thereafter. There were no significant differences in treatments given for local disease in relation to *BRCA* status after matching for tumor receptor status and patient age. Systemic treatments for metastatic disease before CNS metastasis, including chemotherapy, endocrine and HER2-directed treatments and immunotherapy, were also similar. Most patients received systemic treatments for non-CNS metastatic disease, with 34.7% of all patients receiving one line of treatment, and 14.7% receiving four or more systemic treatment lines before the diagnosis of CNS disease.

There were no differences in sites of metastatic disease in relation to *BRCA* status. 13.3% of all patients were diagnosed with CNS metastases as single metastatic site at disease presentation, and 8% presented with CNS metastases along with other distant metastatic sites. Most patients had several non-CNS metastatic sites, although 8% of each group had CNS-only disease during the entire follow-up period.

### Study population comparison after CNS metastasis diagnosis

The characteristics of CNS involvement and CNS-directed treatments given after CNS metastasis diagnosis are listed in Table 2. Mean age at diagnosis of CNS metastasis was 55.0 ±10.4 years (range 26.7–73.9) in the non-*BRCA* mutation group, and 46.5 ±13.1 years (range 30.0–78.3) with *BRCA*1/2 mutation, *P* < 0.0001. More patients with *BRCA* PVs had good performance status (ECOG PS 0) during the diagnosis of brain metastasis (20% vs. 2%, *P* = 0.033), although at this point a high number of patients in both groups presented with functional deterioration as a result of symptoms related to their CNS-disease.

**Table 2:** Clinical and pathological factors and treatment characteristics after CNS metastasis diagnosis according to BRCA mutation status- carriers (BRCA 1/2 Mt) vs non-carriers (BRCA non-Mt)

Characteristics	BRCA non-Mt (*n* = 50)	BRCA 1/2 Mt (*n* = 25)	Total (*n* = 75)	*P-value*
**Age at diagnosis of CNS metastasis (yr)**				
Median	55.3	44.33	53.05	0.003
Mean ± SD	55.02 ± 10.37	46.49 ± 13.05	52.17	
Range	26.71–73.93	30.04–78.26	26.7–78.26	
**ECOG PS at CNS spread**^a^				
ECOS PS 0	1 (2%)	5 (20%)	6 (8%)	0.033
ECOG PS 1	10 (20%)	2 (8%)	12 (16%)	
ECOG PS 2	21 (42%)	6 (24%)	27 (36%)	
ECOG PS 3	14 (28%)	10 (40%)	24 (32%)	
ECOG PS 4	4 (8%)	2 (8%)	6 (8%)	
**Resection of brain metastases**				
Done	11 (22%)	8 (32%)	19 (25.3%)	0.348
Not done	39 (78%)	17 (68%)	56 (74.7%)	
**Brain radiotherapy**^b^				
WBRT	11 (22%)	7 (28%)	18 (24%)	
Partial brain	1 (2%)	0	1 (1.3%)	
SRS	19 (38%)	12 (48%)	31 (41.3%)	
SRS + WBRT	11 (22%)	2 (8%)	13 (17.3%)	
None	8 (16%)	4 (16%)	12 (16%)	
**Location of brain metastases**^c^				
Frontal	33 (66%)	15 (60%)	48 (64%)	0.610
Temporal	13 (26%)	13 (52%)	26 (34.7%)	0.026
Parietal	21 (42%)	10 (40%)	31 (41.3%)	0.868
Occipital	11 (22%)	9 (36%)	20 (26.7%)	0.196
Cerebellar	32 (64%)	13 (52%)	45 (60%)	0.317
Midbrain	5 (10%)	5 (20%)	10 (13.3%)	0.230
Pons	3 (6%)	3 (12%)	6 (8%)	0.367
Medulla	2 (4%)	1 (4%)	3 (4%)	1
**Area of brain metastases**				
Supratentorial only	16 (32%)	10 (40%)	26 (34.7%)	0.890
Infratentorial only	6 (12%)	2 (8%)	8 (10.7%)	
Tumor deposits in both areas	20 (40%)	9 (36%)	29 (38.7%)	
Diffuse spread^d^	8 (16%)	4 (16%)	12 (16%)	
**No. of brain metastases at CNS presentation**				
Single deposit	9 (18%)	7 (28%)	16 (21.3%)	0.093
2–3 deposits	10 (20%)	2 (8%)	12 (16%)	
4–5 deposits	10 (20%)	1 (4%)	11 (14.7%)	
6 or more deposits	4 (8%)	7 (28%)	11 (14.7%)	
Diffuse spread^d^	17 (34%)	8 (32%)	25 (33.3%)	
**Leptomeningeal spread**				
Yes	10 (20%)	10 (40%)	20 (26.7%)	0.020
No	40 (80%)	15 (60%)	55 (73.3%)	
**Chemotherapy- CNS disease**				
Anti-microtubule^e^	12 (24%)	5 (20%)	17 (22.7%)	0.072
Platinum based	9 (18%)	6 (24%)	15 (20%)	
Anthracycline based	7 (14%)	4 (16%)	11 (14.7%)	
Capecitabine	11 (22%)	4 (16%)	15 (20%)	
None	11 (22%)	6 (24%)	17 (22.7%)	
**Hormonal therapy- CNS disease**				
Tamoxifen	3 (6%)	1 (4%)	4 (5.3%)	0.078
AI^f^	5 (10%)	0	5 (6.7%)	
Fulvestrant	4 (8%)	0	4 (5.3%)	
Anti-CDK4/6 ^g^ +/−	6 (12%)	0	6 (8%)	
AI/ Fulvestrant				
None	32 (64%)	24 (96%)	56 (74.7%)	
**anti-HER2- CNS disease**				
Trastuzumab	1 (2%)	1 (4%)	2 (2.7%)	0.246
Trastuzumab+Pertuzumab	0	1 (4%)	1 (1.3%)	
TDM1	6 (12%)	3 (12%)	9 (12%)	
TKI based^h^	8 (16%)	7 (28%)	15 (20%)	
None	35 (70%)	13 (52%)	48 (64%)	
**Immunotherapy post-CNS metastasis**				
Yes	1 (2%)	3 (12%)	4 (5.3%)	0.069
No	49 (98%)	22 (88%)	71 (94.7%)	
**No. treatment lines post- CNS metastasis**				
No systemic treatment	4 (8%)	3 (12%)	7 (9.3%)	0.543
1 line	21 (42%)	15 (60%)	36 (48%)	
2 lines	11 (22%)	4 (16%)	15 (20%)	
3 lines	6 (12%)	1 (4%)	7 (9.3%)	
4 lines or more	8 (16%)	2 (8%)	10 (13.3%)	

Values are n(%) unless otherwise indicated

*ECOG* Eastern Cooperative Oncology Group: *PS* performance status: *CNS* central nervous system: *WBRT* whole-brain radiation therapy: *SRS* stereotactic radiosurgery: *AI* aromatase inhibitor: *CDK* cyclin-dependent kinase: *HER2* human epidermal growth factor receptor: *TDM1* Ado-trastuzumab emtansine: *TKI* tyrosine-kinase inhibitor

^a^ECOG Performance Status, as published in: *Oken, M.M., Creech, R.H., Tormey, D.C., Horton, J., Davis, T.E., McFadden, E.T., Carbone, P.P. Toxicity and response criteria of The Eastern Cooperative Oncology Group. Am J Clin Oncol 5:649–655, 1982*

^b^To test the variable, None/Not done was removed, thus, only the applicable categories were compared

^c^Location of brain metastases- according to CT/MRI at diagnosis of CNS spread. A patient can have more than one site of CNS involvement and therefore can be included in more than one category

^d^Diffuse spread, individual brain deposits cannot be defined

^e^Anti-microtubule based therapy- including: Taxane (Paclitaxel, Docetaxel), Vinorelbine, Eribulin

^f^Aromatase inhibitor- including: non-steroidal (Letrozole, Anastrozole), steroidal (Exemestane)

^g^Anti-CDK4/6 based therapy- including: Palbociclib (non-BRCA Mt: *n* = 4), Ribociclib (*n* = 0), Abemaciclib (non-BRCA Mt: *n* = 2)

^h^TKI based therapy- including: Lapatinib (non-BRCA Mt: *n* = 6, BRCA Mt: *n* = 5), Neratinib (non-BRCA Mt: *n* = 2), Tucatinib (BRCA Mt: *n* = 2)

^i^Immunotherapy post-CNS metastasis- including: Pembrolizumab (non-BRCA Mt: *n* = 1, BRCA Mt: *n* = 3), Atezolizumab (*n* = 0)

There were no significant between-group differences in CNS-directed treatments. Surgical resection of brain metastases was performed in 25.3% of all patients, and 84% of patients received brain radiation therapy (WBRT- whole-brain radiation therapy and/or SRS- stereotactic radiosurgery) at some point after the diagnosis of CNS-disease.

Examining the locations of brain deposits according to brain imaging (CT and/or MRI), a higher incidence of temporal lobe involvement was seen in patients with *BRCA* PVs after matching for tumor receptor status and patient age (52% vs. 26%, *P* = 0.026), with no significant differences for other locations. Most patients in both groups had supra and infra-tentorial disease spread. There was no significant difference in the number of brain deposits in relation to *BRCA* status, and a high number of patients had 6 or more brain deposits or diffuse brain spread, defined as widespread involvement of the brain in which individual brain deposits cannot be defined. In patients with *BRCA* PVs, there was a higher incidence of leptomeningeal spread (40% vs. 20%, *P* = 0.020).

Systemic treatment after diagnosis of CNS disease consisted mainly of chemotherapy in both groups, and 92.7% of all patients received systemic treatment at this point in their course of disease. 13.3% of all patients received 4 of more treatment lines after CNS-spread.

**Fig. 1:**
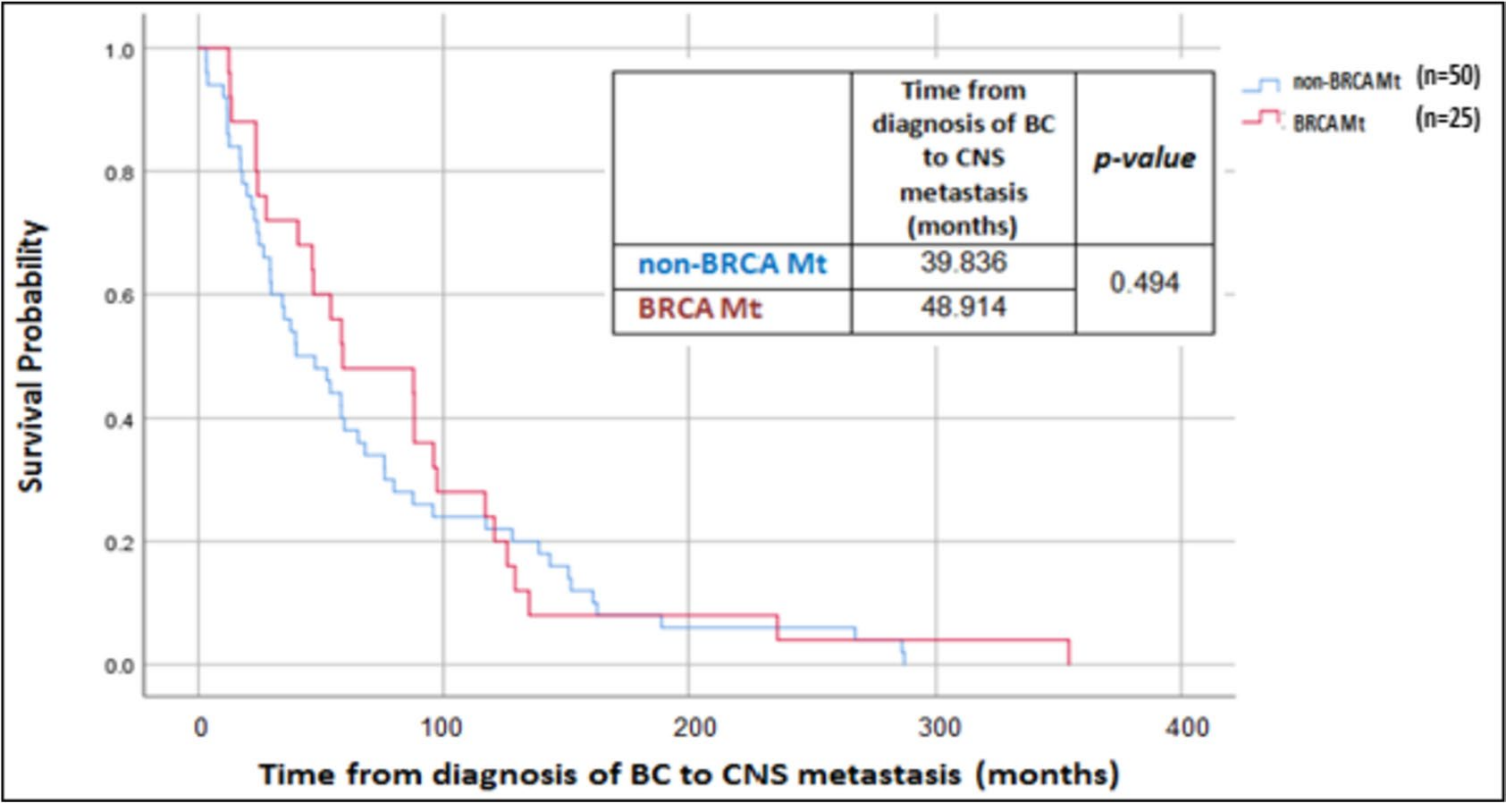
Time from breast cancer (BC) diagnosis to CNS metastasis

**Fig. 2:**
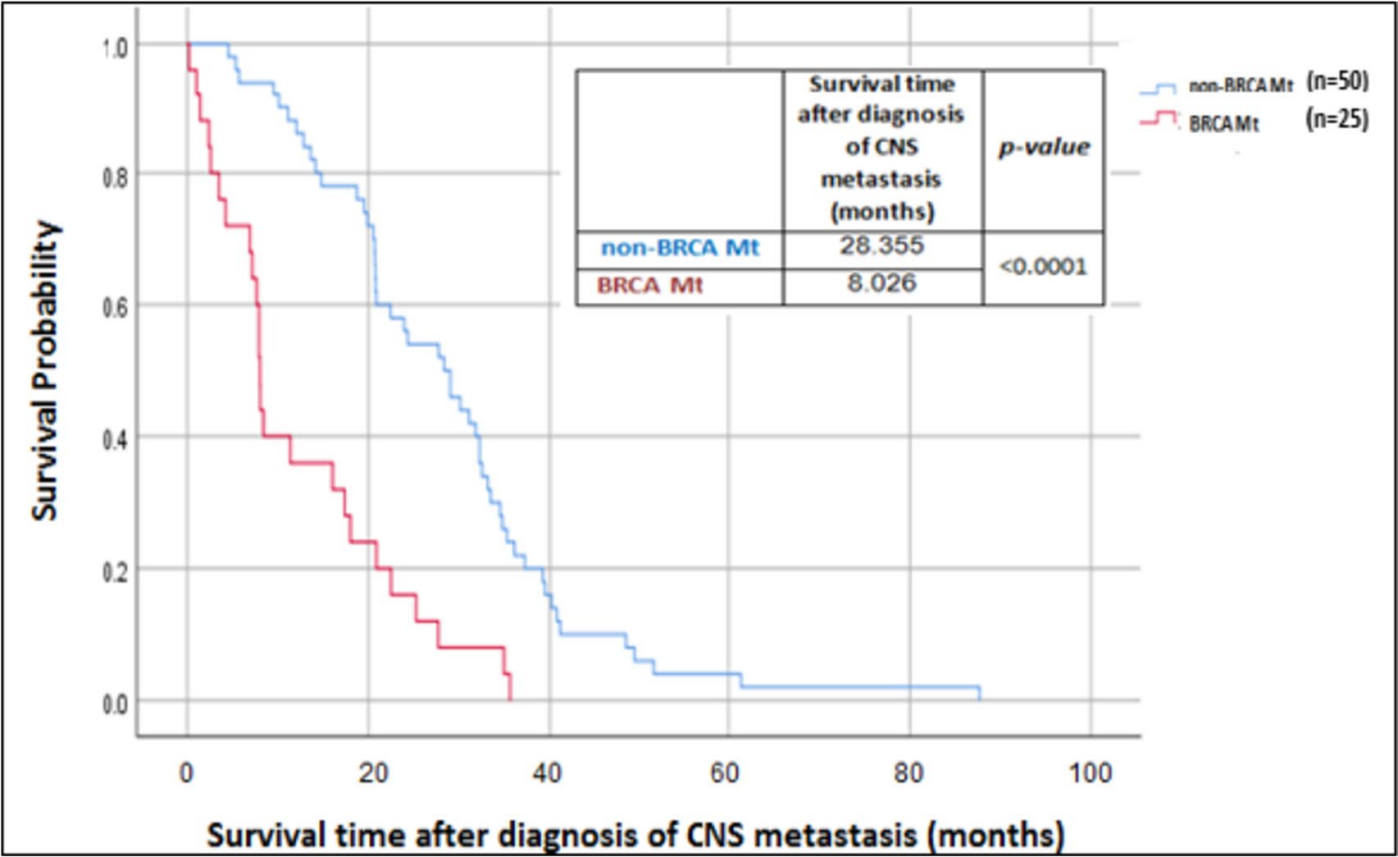
Survival time after diagnosis of CNS metastasis

There was no significant difference in time from breast cancer diagnosis to CNS metastasis in relation to *BRCA* status after matching for tumor receptor status and patient age. Median time for CNS-spread was 39.8 months (range 18.2–61.2 months) in non-*BRCA* mutation patients and 48.9 months (range 3.8–6.2 months) in *BRCA* mutated patients (χ^2^(1) =0.467, *P* = 0.494), see Fig. 1. Survival time after diagnosis of CNS metastasis was shorter in patients with PVs in *BRCA*, with a median survival of 8.0 months (range 7.5–8.5 months), compared to 28.4 months (range 21.2–35.5 months) in *BRCA*-WT patients (χ^2^(1) =23.651, *P* < 0.0001), see Fig. 2.

**Fig. 3:**
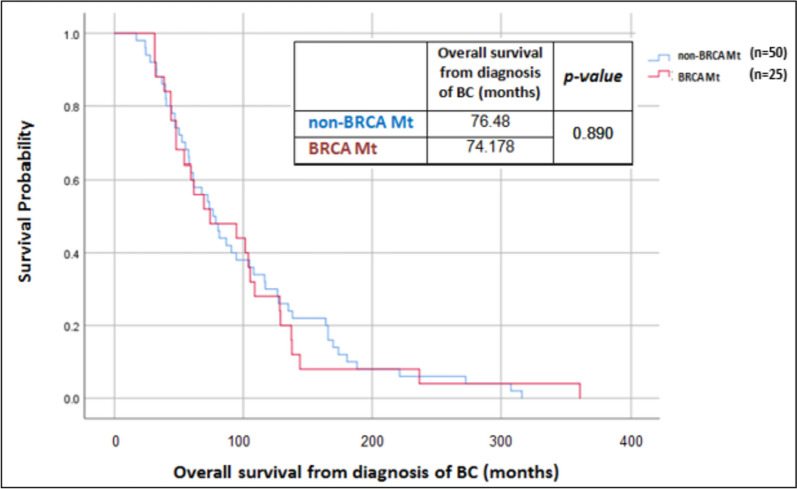
Overall survival from diagnosis of breast cancer (BC)

Median overall survival time from diagnosis of breast cancer to study end or death was 76.5 months (range 60.6–92.4 months) in non-*BRCA* mutated patients and 74.48 months (range 20.06–128.29 months) in *BRCA* mutated patients, with no statistically significant difference between matched groups (χ^2^(1) =0.019, *P* = 0.890), see Fig. 3.

## Discussion

*BRCA* PVs are known to be correlated with younger age at diagnosis of breast cancer- median 38 years in *BRCA*1 mutation and 41 years in *BRCA*2 mutation, as compared to 43 years in non-*BRCA* mutated women, and for *BRCA*1- a higher incidence of high-grade tumors (87%) and triple-negative disease (73%) [15, 16]. Accordingly, in our study, there were more high-grade tumors in *BRCA*-mutated patients (88% vs. 68%, *P* = 0.060). In our former study, we showed that breast cancer patients who were diagnosed at a younger age (< 45 years) developed brain metastases earlier in their course of disease, and for patients with triple-negative disease, a younger age at breast cancer diagnosis was related to a longer survival time after diagnosis of CNS-disease [18]. In light of these findings, we chose to preform our current data analysis controlling for the possible influence of the patient age at breast cancer diagnosis and tumor receptor status (ER, PR, HER2). After matching for age and receptor status, our findings did not suggest an earlier CNS spread in patients with *BRCA*1/2 mutations, and the median time from breast cancer diagnosis to CNS disease was relatively long in both groups.

Leptomeningeal carcinomatosis is a late complication resulting from tumor cells spread in the cerebrospinal fluid (CSF) into the leptomeninges. Studies have shown that 5% of breast cancer patients will develop leptomeningeal disease during the course of disease, mostly at a late stage and in the presence of diffuse metastatic disease in multiple systemic sites [19]. In our study, there was a relatively high incidence of leptomeningeal disease (26.7% of all patients), and in the presence of *BRCA*1/2 mutation, 40% of patients presented with leptomeningeal disease during the follow-up period, as compared to 20% without a *BRCA* mutation (*P* = 0.020). Looking at the high incidence of leptomeningeal disease in our database, one should remember that our study population consisted entirely of women with brain metastases, therefore it is expected that the incidence of leptomeningeal involvement will be higher than the incidence in a population of women with breast cancer. For example, a study examining the incidence of leptomeningeal disease in women with breast cancer treated with SRS, showed that 25% of patients developed leptomeningeal metastasis 1 year after SRS treatment [20]. Leptomeningeal involvement is a negative prognostic factor in breast cancer, with median survival time of only 4.9 months [21]. In our study, there was a shorter survival rate following the diagnosis of CNS-disease in women with *BRCA*1/2 mutation, which could have been influenced by this adverse prognostic factor.

In the time of diagnosis of CNS-disease, patients with *BRCA*1/2 mutation were younger (median age 46.69 years vs. 55.02 years, *P* = 0.003) and in better performance status (ECOG PS 0 in 20% vs. 2%, *P* = 0.033). Earlier studies have shown that performance status at diagnosis of brain metastasis is an important prognostic factor which effects patient survival [8]. In our study, despite of the difference in favor of the *BRCA* mutated group, the survival time after diagnosis of CNS-disease was shorter.

There were no significant differences in the type of local or systemic treatment modalities given before or after CNS-spread, also in the context of treatments which are known to penetrate the blood brain barrier, and therefore can affect the incidence and course of CNS-metastatic disease, such as capecitabine, Ado-trastuzumab emtansine (TDM1), or abemaciclib. As of note, our data were analyzed in an era prior to the regular use of treatments such as new generation HER2-directed therapies (fam-trastuzumab deruxtecan-nxki; ENHERTU) for HER2-positive metastatic disease, and before the use of olaparib (Lynparza) as adjuvant treatment for patients with germline *BRCA*-mutated HER2-negative high-risk early breast cancer who have been treated with chemotherapy before or after surgery.

Examining the characteristics of CNS-spread, after matching for patient age and tumor receptor status, our data show a higher rate of temporal lobe involvement in patients with *BRCA* mutation (52% vs. 26%, *P* = 0.026), with no significant differences for other locations of brain metastases. In a review of literature, only a small number of studies examined possible parameters affecting the locations of CNS-spread in breast cancer, most of them in relation to the influence of tumor receptor status (ER, PR, HER2). A retrospective study of 60 breast cancer patients with brain metastases showed that ER-negative HER2-positive tumors had a higher incidence of multiple brain metastases (6 or more)- 61% vs. 36% in other tumor subtypes (*P* = 0.002), and a higher incidence of brain stem metastasis (61% vs. 11.5%, *P* = 0.002) and occipital metastases (66.7% vs. 30.8%, *P* = 0.029) [22]. A study published as an abstract in 2018 included 50 breast cancer patients with brain metastases, in which ER-positive HER2-positive tumors showed a higher rate of cerebellar metastasis (*P* < 0.05) [23]. Another study examined the MRI scans of 100 breast cancer patients with a known CNS-disease, exhibiting a higher rate of occipital and cerebellar metastasis for HER2-positive and/or ER-positive tumors, and a higher rate of frontal, parietal and limbic region involvement for triple-negative tumors (*P* < 0.05) [24].

The locations of CNS disease spread affect clinical manifestations, with possible prognostic implications. A study published in *BMC cancer* in 2023 showed a higher rate of seizures in metastatic breast cancer patients with BRCA mutation [25], and our results regarding the differences in CNS spread in BRCA mutation carriers can be a possible explanation to these findings. Another study showed that BRCAness in breast cancer patients with brain metastases was associated with shorter overall survival, regardless of clinicopathological factors [26].

Our study was limited by the retrospective and nonrandomized design, with a relatively small sample size and a short follow-up period. Furthermore, the study population reflects the changes in treatment paradigms over time, with advancements made in local and systemic therapies, imaging modalities, and the increasing use of molecular testing to guide individualized patient treatment. Strengths of our study include detailed information available for each patient, such as tumor characteristics during the course of disease, local and systemic treatments, and other important prognostic factors. Patients were treated in a single institute with uniform guidelines for diagnosis, treatment, and follow-up. Additionally, the comparison using matched-pair analysis allowed us to counterbalance the possible influence of other prognostic variables, and therefore to investigate further the unique differences that can be attributed to *BRCA* mutation status.

## Conclusions

In this 1:2 matched-pair analysis of CNS metastatic breast cancer, patients with germline pathogenic variants (PVs) in *BRCA*1/2 genes showed unique features regarding their CNS disease and prognosis, also when controlling for patient age and tumor receptor status (ER, PR, HER2), and despite no apparent differences in other known prognostic factors or clinical management. In the presence of *BRCA*1/2 mutations, there was a higher rate of leptomeningeal and temporal lobe involvement, and a shorter survival after the diagnosis of CNS disease. Further research of a larger number of patients with longer follow-up is warranted.

## Data Availability

The database used in this study can be accessed in the permission of the corresponding author on reasonable request.

## Abbreviations

CNS: central nervous system
PVs: pathogenic variants
ER: estrogen receptor
PR: progesterone receptor
HER2: human epidermal growth factor receptor 2
ECOG: eastern cooperative oncology group
CT: contrast-enhanced computed tomography
MRI: magnetic resonance imaging
VUS: variants of uncertain significance
BRCA- WT: BRCA wild-type
IHC: immunohistochemistry
FISH: fluorescence in situ hybridization
P: Significance level (*P*-value)
CI: confidence level (confidence interval)
IDC: infiltrating ductal carcinoma
ILC: infiltrating lobular carcinoma
SLNBx: sentinel lymph node biopsy
ALND: axillary lymph node dissection
BSO: bilateral salpingo-oophorectomy
LHRH: luteinizing hormone-releasing hormone
WBRT: whole-brain radiation therapy
SRS: stereotactic radiosurgery
CSF: cerebrospinal fluid
TDM1: Ado-trastuzumab emtansine
BC: breast cancer
